# Diurnal regulation of *SDG2* and *JMJ14* by circadian clock oscillators orchestrates histone modification rhythms in *Arabidopsis*

**DOI:** 10.1186/s13059-019-1777-1

**Published:** 2019-08-20

**Authors:** Qingxin Song, Tien-Yu Huang, Helen H. Yu, Atsumi Ando, Paloma Mas, Misook Ha, Z. Jeffrey Chen

**Affiliations:** 1 Department of Molecular Biosciences, The University of Texas at Austin, Austin, TX 78712 USA; 2 Department of Integrative Biology, The University of Texas at Austin, Austin, TX 78712 USA; 3 State Key Laboratory of Crop Genetics and Germplasm Enhancement, Nanjing Agricultural University, Nanjing, 210095 China; 4 Center for Research in Agricultural Genomics (CRAG), Consortium CSIC-IRTA-UAB-UB, Campus UAB, Bellaterra, 08193 Barcelona, Spain; 5 Samsung Advanced Institute of Technology, Samsung Electronics Corporation, Suwon, 443-803 South Korea

**Keywords:** Diurnal, Histone modification, *Arabidopsis*

## Abstract

**Background:**

Circadian rhythms modulate growth and development in all organisms through interlocking transcriptional-translational feedback loops. The transcriptional loop involves chromatin modifications of central circadian oscillators in mammals and plants. However, the molecular basis for rhythmic epigenetic modifications and circadian regulation is poorly understood.

**Results:**

Here we report a feedback relationship between diurnal regulation of circadian clock genes and histone modifications in *Arabidopsis*. On one hand, the circadian oscillators CCA1 and LHY regulate diurnal expression of genes coding for the eraser (JMJ14) directly and writer (SDG2) indirectly for H3K4me3 modification, leading to rhythmic H3K4me3 changes in target genes. On the other hand, expression of circadian oscillator genes including *CCA1* and *LHY* is associated with H3K4me3 levels and decreased in the *sdg2* mutant but increased in the *jmj14* mutant. At the genome-wide level, diurnal rhythms of H3K4me3 and another histone mark H3K9ac are associated with diurnal regulation of 20–30% of the expressed genes. While the majority (86%) of H3K4me3 and H3K9ac target genes overlap, only 13% of morning-phased and 22% of evening-phased genes had both H3K4me3 and H3K9ac peaks, suggesting specific roles of different histone modifications in diurnal gene expression.

**Conclusions:**

Circadian clock genes promote diurnal regulation of *SDG2* and *JMJ14* expression, which in turn regulate rhythmic histone modification dynamics for the clock and its output genes. This reciprocal regulatory module between chromatin modifiers and circadian clock oscillators orchestrates diurnal gene expression that governs plant growth and development.

**Electronic supplementary material:**

The online version of this article (10.1186/s13059-019-1777-1) contains supplementary material, which is available to authorized users.

## Background

Circadian clocks anticipate diurnal cycles to coordinate physiological and developmental processes in all organisms from cyanobacteria, flies, to humans and plants [1–3], which are achieved through feedback transcriptional-translational regulatory modules [4–7]. In the transcriptional module, chromatin modifications regulate central circadian oscillators in mammals [8–10] and plants [7, 11–14]. In mouse liver, there are time-dependent patterns of transcription factor binding, RNA polymerase II recruitment, RNA abundance, and chromatin states, which account for 22% of mRNA cycling genes, suggesting a transcriptional mechanism for regulating the mammal circadian network [8].

In *Arabidopsis*, the central transcriptional loop involves two morning-phased Myb-like transcription factors, CIRCADIAN CLOCK ASSOCIATED 1 (CCA1) and LATE ELONGATED HYPOCOTYL (LHY) [15, 16], which repress the evening-phased regulator, *timing of CAB expression 1* (*TOC1*), by directly binding to its promoter [17, 18]. In turn, TOC1 indirectly promotes the expression of *CCA1* and *LHY* by inhibiting the repressive *PSEUDO-RESPONSE REGULATOR* (*PRR*) genes or directly represses expression of *CCA1* and *LHY* by binding their promoters [19, 20]. Integrating other clock regulators including GI and ELF4–ELF3–LUX complex with the central CCA1/LHY/TOC1 loop provides a classic feedback regulatory mechanism for the circadian clock in *Arabidopsis* [21, 22], which includes a number of core molecular oscillators that constitute interlocking transcriptional-translational negative feedback loops (see reviews in [2, 4, 7, 23, 24]).

Using candidate gene approaches, several studies have indicated links between histone modifications and expression of circadian clock genes. For example, induction of the morning-phased *TOC1* expression wave form is related to histone acetylation, which is repressed by CCA1 and coincident with histone deacetylase activities and repressive chromatin status at dawn [12]. Rhythmic expression of *CCA1* and *LHY* and their reciprocal regulator *TOC1* is associated with H3K4me3 and H3K9/14 ac levels in the translational start sites of these genes [11, 13], but correlated negatively with H3K36me2 levels [11]. Moreover, changes in diurnal transcript levels correlate with H3K9ac, H3K27ac, and H3S28p levels between end-of-day and end-of-night [25]. Inhibiting acetylation and H3K4me3 abolishes rhythmic expression of circadian oscillators, while blocking H3K4me3 leads to increased levels of clock-repressor binding, suggesting a transitional role for the H3K4me3 mark in modulating clock gene expression from activation to repression [14]. JMJD5, a histone demethylase in humans and plants, is co-regulated with evening-phased clock regulators and promotes expression of clock genes at dawn [26]. However, neither the basis for histone mark rhythms nor the relationship between histone modifications and circadian clock regulators is clearly defined. Here we report dynamic interactions between chromatin modification of circadian clock gene expression and diurnal regulation of histone modifications, which regulate diurnal gene expression networks in *Arabidopsis*.

## Results

### Diurnal regulation of histone methyltransferase and demethylase by clock genes

**Fig. 1:**
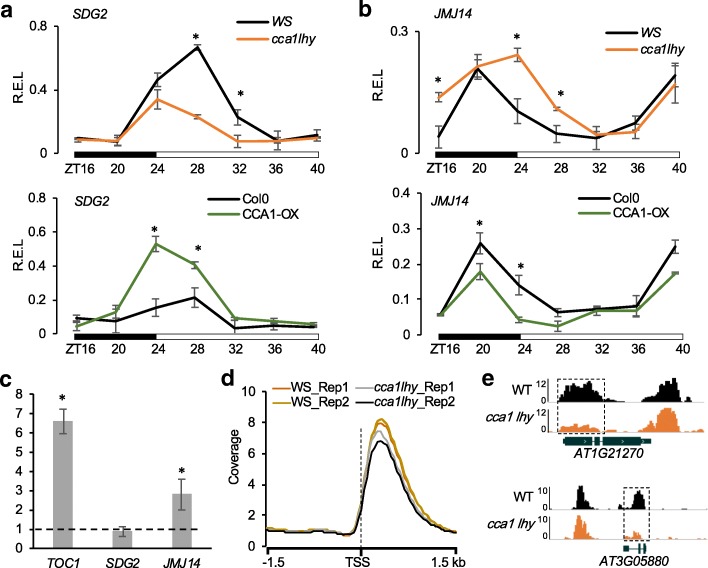
The clock regulates expression of histone methyltransferase (SDG2) and demethylase (JMJ14) genes. **a**, **b** Relative expression levels (R.E.L.) of *SDG2* (**a**) and *JMJ14* (**b**) in the wild type (WT, Ws), *cca1 lhy* mutant (Ws) (upper panel), and WT (Col-0) and CCA1-OX (Col-0) (lower panel) plants under a diurnal cycle (16 h light/8 h dark). Black and white bars indicate dark (ZT16, 20, and 24) and light (ZT28, 32, 36, and 40), respectively. **c** ChIP-qPCR showed the fold enrichment (*Y*-axis) of CCA1-binding fractions in the promoters of *SDG2* and *JMJ14* using rosette tissues from 3-week-old *A. thaliana* at ZT0. The *TOC1* promoter containing evening elements was used as the positive control. *UBQ10* was used as a nonbinding control for normalization. Error bars in **a**–**c** indicate standard deviation of biological replicates (*n* = 3) with an asterisk showing the statistical significance level (*P* < 0.05, Student *t* test). **d** Binding profiles of H3K4me3 at the TSS ± 1.5 kb in the wild type and *cca1 lhy* mutant with two biological replicates. **e** Examples showing decreased binding of H3K4me3 to the genes *AT1G21270* (upper panel) and *AT3G05880* (lower panel) in *cca1 lhy* mutant compared with the WT (Ws)

The association of rhythmic histone modifications with expression of individual circadian clock genes in *Arabidopsis thaliana* [14] and with altered expression of the core clock genes in *Arabidopsis* hybrids and allopolyploids [27] may suggest a general role of histone modifications in diurnal regulation of the circadian clock and its output genes. The diurnal rhythms are likely established through diurnal transcriptional regulation of corresponding genes or diurnal oscillation of histone-modifying enzymes. But the relationship between circadian clock regulators and histone-modifying factors is largely unknown. In *Arabidopsis*, five histone methyltransferases (ATX1, SDG2, SDG4, SDG25, and SDG26) and three histone demethylases (JMJ14, JMJ15, and JMJ18) are reported to be responsible for adding and removing H3K4me3, respectively [28–35]. Among them, SDG2 and JMJ14 are associated with dramatic changes of genome-wide H3K4me3 levels in their respective mutants [36–38], suggesting that these two genes may be primary targets of the circadian clock regulation. To test this, we examined expression patterns of *SDG2* and *JMJ14* subjected to diurnal (24 h, 16 h light/8 h dark) and circadian conditions (48 h, constant light) in the wild type (Col-0). *SDG2* showed rhythmic expression peaks under diurnal but not constant light conditions (Additional file 1: Figure S1a). However, *JMJ14* showed rhythmic expression patterns under both diurnal and constant light conditions (Additional file 1: Figure S1b). Thus, expression patterns of *SDG2* and *JMJ14* are diurnal and circadian, respectively. To further explore the roles of clock genes in *SDG2* and *JMJ14* regulation, we examined *SDG2* and *JMJ14* expression changes under diurnal (24 h, 16 h light/8 h dark, LD) conditions in the wild type (Ws) compared to the *cca1 lhy* double mutant (Ws background) and in the *CCA1* overexpression line (*CCA1-OX*, Col-0 background) relative to Col-0. Under diurnal condition, the expression of *SDG2* peaked in the morning (ZT28, ZT0 or 24 = dawn) in the wild type and was significantly downregulated in the *cca1 lhy* mutant but upregulated in the *CCA1-OX* line (Fig. 1a). Expression of *SDG2* in the WT started to increase from ZT24 (dawn), and overexpressing *CCA1* may have a “burst” effect at ZT24 and accelerated the *SDG2* expression peak (Fig. 1a), with a caveat of a potential genotypic effect (Ws vs. Col).

In contrast to *SDG2*, *JMJ14* expression peaked at night (ZT40) and reached the valley after dawn (ZT28) in the WT and was significantly upregulated in the *cca1 lhy* mutant and downregulated in the *CCA1-OX* line under diurnal condition (Fig. 1b). The data suggest that CCA1/LHY exerts temporal effects on positive and negative correlation, respectively, with *SDG2* and *JMJ14* expression levels. Although both *SDG2* and *JMJ14* have one CCA1-binding site (CBS) in their promoter sequences, *SDG2* and *JMJ14* were not reported as target genes of CCA1 according to the published dataset of chromatin immunoprecipitation sequencing (ChIP-seq), which was performed using CCA1p::CCA1-GFP transgenic plants at ZT2 and ZT14 [39]. To determine whether CCA1 can bind promoters of *SDG2* and *JMJ14* at different time points, we performed ChIP-qPCR using antibodies against CCA1 at ZT0 when CCA expression peaks. ChIP-qPCR showed significant enrichment of CCA1 in the promoters of *JMJ14* and *TOC1* (normalized to *UBQ10*), but not in the promoter of *SDG2* (Fig. 1c). Although we could not exclude a possibility of non-specific binding of CCA1-antibodies, this result indicates that CCA1/LHY may directly bind to the *JMJ14* promoter and regulates its expression, as other members (*JMJ30* and *JMJD5*) of the gene family that are co-regulated with evening-phased genes [26, 40], but may indirectly regulate *SDG2* expression through other factors such as CCA1-mediated genes or in the CCA1 complex. Alternatively, regulation of *SDG2* by CCA1 could occur at a different time of the day. In addition, other H3K4 methyltransferase *ATX1* and H3K4 demethylase *JMJ15* were also down- and upregulated, respectively, in the *cca1 lhy* mutant (Additional file 1: Figure S1c, d). The data suggest that CCA1/LHY can upregulate expression of H3K4me3 writers (SDG2 and ATX1) and downregulate H3K4me3 erasers (JMJ14 and JMJ15), which would result in the overall decreased distributions of H3K4me3 in the *cca1 lhy* mutant.

Using ChIP-seq analysis with antibodies against H3K4me3, we analyzed H3K4me3 distribution patterns in the rosette leaves of wild type (Ws) and *cca1 lhy* mutant plants at dawn (ZT0) under diurnal conditions, when CCA1/LHY expression level peaked [41]. Compared to the wild type, H3K4me3 levels near the transcription start sites of genic regions were damped in the *cca1 lhy* mutant (Fig. 1d). A total of 880 genes showed decreased levels of H3K4me3 peaks (one-way ANOVA, *P* < 0.05) (Additional file 2: Table S1), including two examples shown in Fig. 1e. A previous study has identified CCA1 target genes by ChIP-seq using 2-week-old seedlings grown under 12 h light/12 h dark for 12 days and then transferred to constant light for 2 days [39]. The overlapping fraction is small between the CCA1 target genes (5%, 78/1433) and those of reduced H3K4me3 levels in the *cca1 lhy* mutant (*P* > 0.5, hypergeometric test). The results indicate that CCA1 affects overall H3K4me3 accumulation levels but does not directly participate in the establishment of H3K4me3 marks for CCA1 target genes. However, different growth conditions and tissue stages in these two studies may also contribute to the small overlapping fraction between the CCA1 target genes and genes with reduced H3K4me3 levels in the *cca1 lhy* mutant.

### Disrupted histone modifications lead to altered expressions of clock genes

**Fig. 2:**
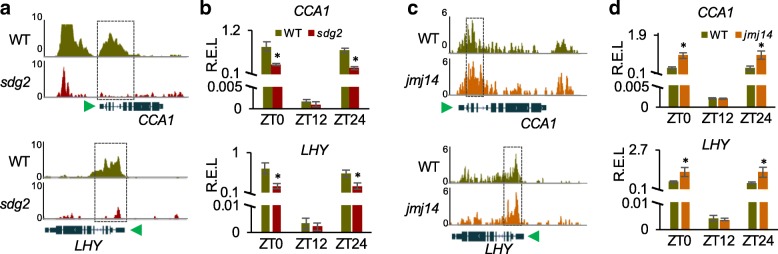
Disrupting histone modifications affects expression of key circadian clock regulators. **a** ChIP-seq assays showing decreased binding of H3K4me3 to the genic regions of *CCA1* (top) and *LHY* (bottom) in the *sdg2* mutant compared with the wild type (WT). Green arrows next to the gene diagrams indicate transcriptional direction. **b** Relative expression levels (R.E.L.) of *CCA1* (top) and *LHY* (bottom) in the *sdg2* mutant compared with the WT at ZT0, ZT12, and ZT24 under a diurnal cycle (16 h light/8 h dark). *Y*-axis was broken as a result from large variation of expression levels. **c** ChIP-seq assays showing increased binding of H3K4me3 to the genic regions of *CCA1* (top) and *LHY* (bottom) in the *jmj14* mutant compared with the WT. **d** R.E.L of *CCA1* (upper) and *LHY* (lower) in the *jmj14* mutant compared with the WT under a diurnal cycle (16 h light/8 h dark). Error bars in **b** and **d** indicate standard deviation of biological replicates (*n* = 3) with an asterisk showing the statistical significance level (*P* < 0.05, Student *t* test)

To test how histone modifications affect expression of circadian clock genes, we analyzed H3K4me3 level changes of the clock genes in the *sdg2* (10-day-old seedlings under long-day conditions) or *jmj14* (3-week-old rosette leaves 16 h light/8 h dark) mutant using the published ChIP-seq datasets [42, 43]. Consistent with the previous report [14], disruption of *SDG2* resulted in a dramatic decrease of H3K4me3 accumulation levels in most clock genes examined, including *CCA1* and *LHY* (Fig. 2a), *TOC1*, *PRR5*, *PRR7*, *PRR9*, *GI*, *ELF3*, *ELF4*, and *LUX* (Additional file 1: Figure S2). ChIP-qPCR using rosette leaves at ZT0, ZT12, and ZT24 further confirmed a significant reduction of H3K4me3 levels near the 5′ region of *CCA1* and *LHY* in the *sdg2* mutant (Additional file 1: Figure S3a). Consequently, expression levels of *CCA1* and *LHY* were dramatically reduced in the *sdg2* mutant (Fig. 2b). In contrast to the results in the *sdg2* mutant, H3K4me3 accumulation levels of *CCA1*, *LHY*, and several other clock genes were slightly increased in the *jmj14* mutant (Fig. 2c and Additional file 1: Figure S2). This moderate effect may be related to the genetic redundancy of JMJ demethylase family including six other members (*JMJ15*, *JMJ16*, *JMJ17*, *JMJ18*, *JMJ19*, and *JMJ30*) in *Arabidopsis* [32, 34, 40]. ChIP-qPCR confirmed a significant increase of H3K4me3 levels near the 5′ region of *CCA1* and *LHY* in the *jmj14* mutant (Additional file 1: Figure S3b), which correlated with increased expression levels of those clock genes (Fig. 2d).

### Dynamic diurnal rhythms of histone modifications in *Arabidopsis*

**Fig. 3:**
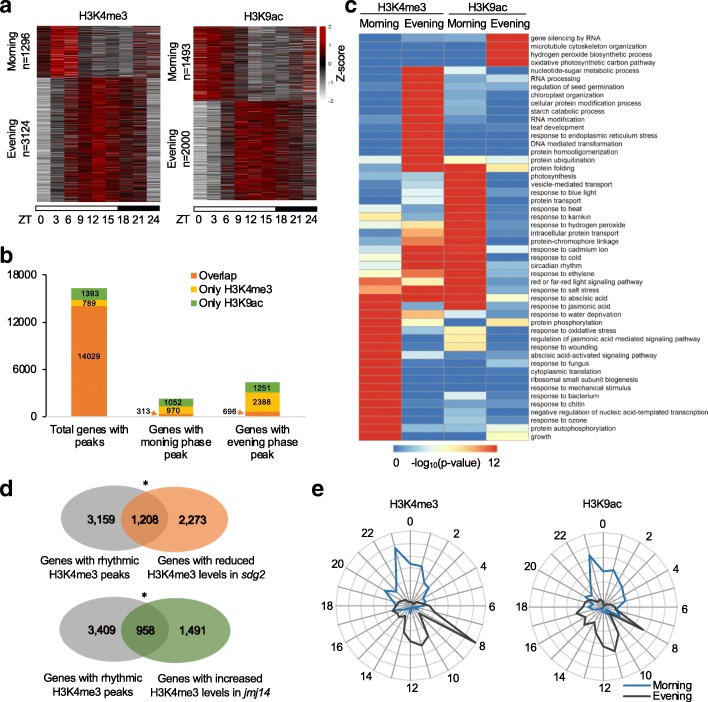
Dynamics of diurnal histone modifications in *Arabidopsis*. **a** Heat-map view of H3K4me3 and H3K9ac binding signals every 3 h in a 24-h period (16 h light/8 h dark). The peaks are partitioned into the morning and evening clusters. The number of peaks in each cluster is indicated at the left side of the panel. **b** Overlap of genes with H3K4me3 and H3K9ac peaks. **c** Gene Ontology (GO) analysis of the genes with diurnal H3K4me3 or H3K9ac binding peaks. **d** Overlap between rhythmic H3K4me3 peaks and peaks with altered H3K4me3 enrichment in the *sdg2* or *jmj14* mutant compared with the WT. The asterisk indicates the statistical significance level for overlap with *P* < 0.05. **e** Phase distributions of the genes with rhythmic H3K4me3 or H3K9ac peaks in the morning phase (blue line) and evening phase (black line)

In animals and plants, histone marks like H3K4me3 and H3K9ac are associated with active transcription and can sometimes be part of transcriptional activation [44, 45]. Clock-mediated diurnal expression of histone-modifying genes suggest rhythmic distributions of histone marks. To examine diurnal rhythms of histone modifications, we used ChIP-seq to examine diurnal rhythms of genome-wide H3K4me3 levels every 3 h in a 24-h period using rosette leaves of *A. thaliana* grown under the long-day cycle (16 h light/8 h dark). For comparative studies, we also performed ChIP-seq experiments using another active histone mark H3K9ac. We identified a total of 15,151 H3K4me3 and 16,966 H3K9ac high-confidence peaks (*P*_adj_ < 0.01, MACS), corresponding to 14,818 and 15,422 unique genes, respectively (Additional file 3: Table S2). The distribution of H3K4me3 and H3K9ac in both evening and morning phases may suggest that these marks are also required for general transcriptional regulation [46]. Of those peaks, 4420 (29%) H3K4me3 and 3493 (21%) H3K9ac peaks exhibited diurnal rhythms at a statistically significant level (*P*_adj_ < 0.01, JTK_CYCLE using Kendall’s tau correlation and Bonferroni correction to adjust *P* value) (Fig. 3a and Additional file 4: Table S3). Those oscillating H3K4me3 and H3K9ac peaks overlapped 4367 and 3276 genes, respectively. This distribution rhythm is not likely affected by histone H3, and only 21 (0.5%) H3K4me3 and 32 (0.9%) H3K9ac rhythmic peaks exhibited histone H3 distribution rhythms at a statistically significant level (*P*_adj_ < 0.01, JTK_CYCLE) (Additional file 1: Figure S3c, d). Another study identified 657 and 1495 genes with oscillating H3K4me3 and H3K9ac peaks, respectively, using two time points (end-of-day and end-of-night) in the short-day condition [25]. Between two datasets, 38% (250/657) and 28% (418/1495) of the genes with rhythmic H3K4me3 and H3K9ac peaks also showed oscillating H3K4me3 and H3K9ac rhythms in our study (*P* < 1e−38, hypergeometric test). The difference between these two studies could be related to the differences in the time points (nine vs. two) examined and in the growth conditions (long-day vs short-day) used between two experiments.

The phase distribution analysis indicated genome-wide binding rhythms of H3K4me3 with a major peak at ZT12 and a minor peak at ZT6, whereas the major peak of H3K9ac binding rhythms was delayed towards ZT15 and the minor peak was moved forward to ZT3 (Additional file 1: Figure S4a). This is consistent with total levels of H3K4me3 and H3K9ac as determined by the Western blot analysis (Additional file 1: Figure S4b). The ChIP-seq data further showed a diurnal rhythm of the total H3K4me3 levels with the peak at ZT12, whereas the total H3K9ac levels did not show obvious peaks during the day and night (Additional file 1: Figure S4b). The inconsistency between diurnal H3K9ac distribution and global H3K9ac content may be related to inconspicuous H3K9ac distribution variation in the time points examined in total H3K9ac levels (ZT0, 6, 12, 18, 24). Alternatively, non-rhythmic H3K9ac distribution may account for the majority of overall H3K9ac variation because of its role in other aspects of plant growth and development [47, 48].

The ChIP-seq data were further classified into the morning-phased (between ZT0 and ZT6) and evening-phased (between ZT9 and ZT18) peaks using k-medoids clustering [49] (Fig. 3a and Additional file 4: Table S3). A majority (86%, 14,029/16,391) of H3K4me3- or H3K9ac-enriched genes had both H3K4me3 and H3K9ac peaks, and only a small proportion of genes was associated with only one histone mark (Fig. 3b). This suggests a coordinated mechanism for H3K4me3 and H3K9ac to regulate gene expression [45]. However, among morning-phased genes, only 13% (313/2335) were associated with both morning-phased H3K4me3 and H3K9ac peaks. Among evening-phased genes, 696 genes (22%) were related to both evening-phased H3K4me3 and H3K9ac peaks (Fig. 3b). The proportion of genes associated with both histone marks in morning-phased (13%) or evening-phased (22%) peaks was significantly lower than that enriched with either H3K4me3 or H3K9ac peaks (*P* < 2e−100, hypergeometric test).

Gene ontology (GO) analysis suggested that the morning- and evening-phased H3K4me3 and H3K9ac marks were enriched in genes with different gene ontology (GO) groups (Fig. 3c). The morning-phased genes are enriched with photosynthetic activities and responses to a variety of stresses, while the evening-phased genes are enriched with DNA and RNA metabolism and protein ubiquitination and folding (Fig. 3c). These data implied a specificity for diurnal H3K4me3 and H3K9ac rhythms in the expression of genes involved in different biological networks. A total of 22% (958/4367) and 28% (1208/4367) genes with rhythmic H3K4me3 peaks in the *sdg2* and *jmj14* mutants, respectively, showed altered H3K4me3 levels (Fig. 3d), which was 1.5-fold higher than those genes (12%, 1252/10,503 and 17%, 1810/10,503) with non-rhythmic H3K4me3 peaks in the mutants, respectively, displaying altered H3K4me3 levels (*P* < 1e−10, Fisher’s exact test). The data suggest that the genes with rhythmic histone modifications are more sensitive to the loss of histone methyltransferase and demethylase activities than other genes.

To examine the correlation between oscillating histone modifications and gene expression, we analyzed expression patterns of genes using published data of expression profiling in *Arabidopsis* under diurnal (16 h light/8 h dark) and circadian (constant light) conditions [41]. For the genes with both diurnal and circadian expression patterns, 14% (612/4377) had both rhythmic H3K4me3 and H3K9ac marks (Additional file 4: Table S3). However, the fraction of both rhythmic H3K4me3 and H3K9ac marks was much lower for the genes with circadian but not diurnal expression patterns (4%, 150/4134) or for the genes with diurnal but not circadian expression patterns (4%, 139/3121). These results suggest different impact of histone-modifying enzymes in diurnal and/or circadian expression patterns. JMJ proteins are circadian-mediated probably through coregulation with the clock components [26, 40], while *SGD2* expression is indirectly regulated by *CCA1* (Fig. 1c and Additional file 1: Figure S1). At the genome-wide level, ~ 48% (2104/4367) of the genes with oscillating H3K4me3 and ~ 47% (1552/3276) of the genes with oscillating H3K9ac displayed rhythmic expression with distinct morning-phased and evening-phased patterns [41] (Fig. 3e), indicating a strong correlation between diurnal histone modifications and rhythmic gene expression.

### Roles of histone modifications in core clock genes

**Fig. 4:**
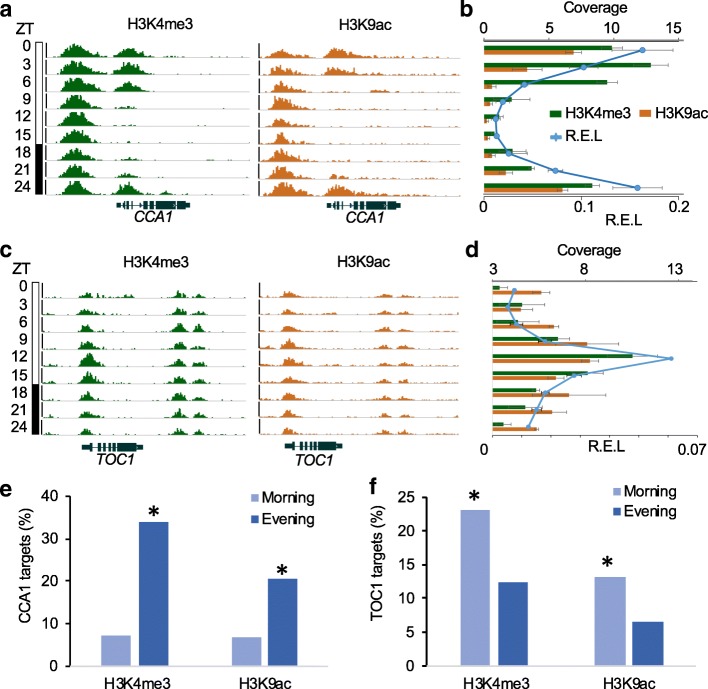
Histone modifications mediate regulation of the clock genes. **a**, **c** Binding signals of H3K4me3 and H3K9ac at the genic regions of *CCA1* (**a**) and *TOC1* (**c**) at different time points. Each track represents the normalized ChIP-seq read coverage at a single time point. The *Y*-axis scales are 0–15 for H3K4me3 and 0–10 for H3K9ac. ZT time is indicated on the left. **b**, **d** Quantifications of the ChIP-seq signals at diurnal peaks located in *CCA1* (**b**) and *TOC1* (**d**), respectively. Relative expression levels (R.E.L.) of *CCA1* and *TOC1* are shown (blue line). *ACT7* was used as an internal control for gene expression. **e**, **f** Enrichment of *CCA1* (**e**) and *TOC1* (**f**) binding targets in the genes with rhythmic H3K4me3 or H3K9ac peaks. Error bars indicate the standard deviation of biological replicates (*n* = 3) with an asterisk showing the significant enrichment (*P* < 1e−5, hypergeometric test)

Further analysis of the ChIP-seq data showed rhythmic patterns of either or both H3K4me3 and H3K9ac marks for all core clock genes examined, including *CCA1*, *LHY*, *TOC1*, *PRR5*, *PRR7*, *GI*, *ELF3*, and *LUX* (Fig. 4a–d and Additional file 1: Figure S5 and S6). This confirms previous reports about the correlation between oscillating chromatin modifications and rhythmic expressions of key clock genes, including *LHY*, *CCA1*, *TOC1*, *PRRs*, and *LUX* [11–14]. Most core clock genes possessed both marks; the morning-expressed genes like *CCA1* had H3K4me3 and H3K9ac peaks at dawn (ZT0-ZT3) (Fig. 4a), while the evening-expressed genes like *TOC1* had H3K4me3 and H3K9ac peaks at noon (ZT12) (Fig. 4c). Expectedly, the transcript levels of *CCA1* and *TOC1* correlated strongly with H3K4me3 and H3K9ac accumulation on a diurnal basis (Fig. 4b, d). This suggests a synchronization between circadian expression and chromatin rhythms.

A possibility for expression rhythms and chromatin modifications is that the core circadian clock genes may interact with chromatin-modifying enzymes to alter histone modifications and regulate target gene expression. To test this, we analyzed binding targets of CCA1 and TOC1 from the published ChIP-seq datasets [20, 39], and examined enrichment levels of H3K4me3 and H3K9ac in the target genes. As CCA1 and LHY often repress expression of evening-phased genes by binding their promoters [39], the majority of CCA1 targets showed rhythmic expression patterns with an evening phase. The results showed that approximately 33% and 20% of the CCA1 targets had evening-phased binding patterns for H3K4me3 and H3K9ac (*P* < 1e−50, hypergeometric test), respectively, while fewer than 7% of CCA1 targets exhibited morning-phased profiles for H3K4me3 and H3K9ac (Fig. 4e). This finding is consistent with the expression patterns of those CCA1 target genes in the evening phase (Additional file 1: Figure S7), although phased distributions of morning and evening genes could change under long- or short-day conditions [41, 50]. In contrast, 23% and 13% of TOC1 target genes correlated with H3K4me3 and H3K9ac marks in the morning phase (Fig. 4f) (*P* < 1e−5, hypergeometric test), while a smaller proportion of target genes correlated with those marks in the evening. This is consistent with TOC1 being a regulator of the morning-phased genes [19, 20] (Additional file 1: Figure S7). To further test the roles of SDG2 and JMJ14 in circadian-mediated gene expression, we randomly selected three CCA1 target genes and examined enrichment of H3K4me3 in genic region of these genes in wild type and *sdg2* and *jmj14* mutants at ZT0, 12, and 24. Enrichment of H3K4me3 in all three CCA1 target genes was significantly decreased in the *sdg2* mutant at similar levels in the time points tested but increased in the *jmj14* mutant in most time points (Additional file 1: Figure S8). The effect in a few time points was moderate in the *jmj14*, probably due to genetic redundancy, as observed for the clock genes (Fig. 2c and Additional file 1: Figure S3). Notably, the H3K4me3 levels in the *sdg2* mutant are reduced at all time points tested (Additional file 1: Figure S8a), consistent with that *SDG2* is not directly regulated by CCA1 (Fig. 1c). JMJ14, however, showed increased H3K4me3 levels with relative rhythms (Additional file 1: Figure S8b), consistent with a role for JMJ proteins in co-regulating with the circadian clock and its target genes [26, 32]. These data indicate that SDG2 and JMJ14 are involved in regulation of rhythmic deposition of H3K4me3 in CCA1 target genes.

## Discussion and conclusions

**Fig. 5:**
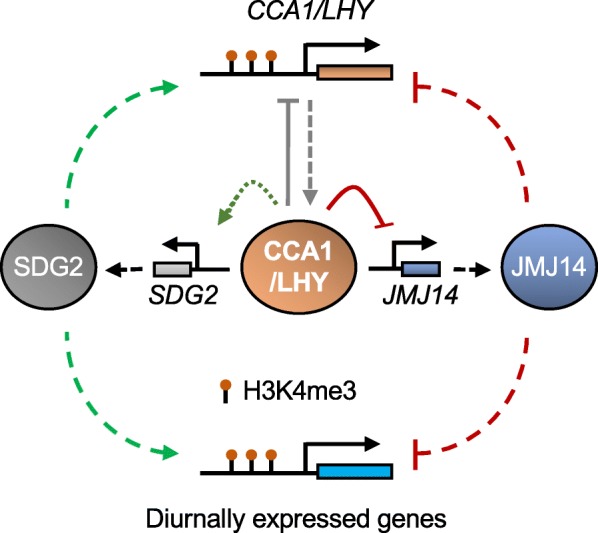
Histone modifications regulate expression of key circadian clock regulators. A model showing feedback regulation between histone H3K4 trimethylation (H3K4me3) and circadian clock regulation through interactions with central clock oscillators (CCA1/LHY) and H3K4me3 writers such as SDG2 and erasers such as JMJ14. Solid lines demonstrated direct effects of positive (arrow) and negative (stop) interactions between CCA1 and JMJ14 (red) and SDG2 (green); dashed lines proposed indirect effects of positive (arrow) and negative (stop) interactions between CCA1 and JMJ14 (red) and SDG2 (green). CCA1/LHY can form homodimers for autoregulation (gray lines) in positive (arrow) and negative (stop) directions

We provided a comprehensive genome-wide landscape of histone modification dynamics in *Arabidopsis*, which has supported diurnal regulation of histone modifications on several individual circadian clock genes as previously reported [11–14, 25, 26]. The current data support a model that integrates clock rhythms with histone modifications to establish rhythmic activities of gene expression, growth, and development (Fig. 5). Circadian clock genes *CCA1* and *LHY* correlate indirectly with the upregulation of the histone methyltransferase gene *SDG2* and directly with repression of the histone demethylase gene *JMJ14* to coordinate histone modification levels in the downstream genes of the circadian output network. Coincidentally, recent studies showed REVEILLE8 interacted with NIGHT LIGHT-INDUCIBLE AND CLOCKREGULATED proteins (LNKs) [51–53] to regulate RNA Pol II and H3K4me3 occupancy on *PRR5* and *TOC1* loci to control their expression [54], further confirming the complex interaction between circadian oscillator, histone modifications, and basal transcriptional machinery. It is predictable that the dynamic histone modifications could change the chromatin structures of genes and affect the accessibility of clock regulators and/or RNA Polymerase II, contributing to rhythmic expression of the target genes [7, 8]. On the other hand, histone methyltransferases and demethylases directly regulate the expression of core clock genes through adding or removing histone modifications. This interactive and reciprocal regulation between circadian clock oscillators and chromatin modifiers orchestrates diurnal gene expression that controls growth and development in plants and animals [6, 55].

A large proportion of histone modification sites exhibit rhythmic patterns, while different histone modifications (H3K4me3 and H3K9ac) have distinct phases for their target genes, which is consistent with the report in mammals [8]. The data collectively indicate a conserved correlation between histone modifications and circadian clock regulation in plants and mammals, as well as a specific distribution of different histone modifications in morning-phased and evening-phased genes. This could be related to distinct peaks of H3K4me3 and H3K9ac in the morning and evening phases, respectively, which correspond to expression of morning- and evening-phased genes (Fig. 3). We also noticed both H3K4me3 and H3K9ac showed lower levels at the night (ZT18-24) than during the day (Fig. 3a). One possibility is that total RNA transcription is lower at night than during the day, resulting in lower levels of H3K4me3 and H3K9ac modifications at night. Alternatively, activation of those genes at the night may not require both H3K4me3 and H3K9ac modifications. Other histone modifications could also be involved in evening-phased genes. Further examination of additional histone marks will provide a comprehensive view for diurnal rhythms of histone modifications and gene expression.

For H3K4me3, the rhythm for regulating circadian-mediated gene expression is associated with expression of the H3K4me3 methyltransferase gene *SDG2*, which is expressed at significantly higher levels during the day than at night; on the other hand, the expression level of the H3K4me3 demethylase gene (*JMJ14*) is low during the day but high at night. This provides a basis for transcriptional regulation of rhythmic histone modifications. Indeed, both global H3K4me3 and morning-phased H3K4me3 levels peak during the day (ZT12) (Additional file 1: Figure S4), suggesting that H3K4me3 is largely established during the day. However, the global H3K4me3 peak is delayed 8 h, compared to the expression peak of H3K4me3 methyltransferase *SDG2* (ZT4). This could be related to the protein and mRNA that accumulate at different levels in a diurnal cycle. For example, CCA1 binds to the *JMJ14* promoter at ZT0 in our ChIP-qPCR study but *JMJ14* is not a target gene at ZT2 or ZT14 in the ChIP-seq analysis [39]. CCA1 protein peaks 3 h later than mRNA in maize [56]. The decrease of *JMJ14* transcripts during the day period may result in the presence of H3K4me3 for a longer time. In addition, the dynamic histone modifications depend on maximal activity of histone-modifying enzymes that may change over time. Notably, other members of H3K4me3 writers (e.g., ATX1) and erasers (e.g., JMJ15) may also contribute to expression rhythms of circadian-mediated regulatory networks.

How histone methyltransferases and histone demethylases are recruited to their target genes remains unknown. One possibility is that histone methyltransferases such as ATX1 can be recruited by the transcriptional machinery through interaction with the Ser5-phosphorylated CTD of Pol II for transcription initiation and elongation [46]. Alternatively, histone modifications could be established through the interaction of sequence-specific transcription factors with histone methyltransferases/demethylases to regulate target gene expression [57, 58]. The targeting and binding time of transcription regulators for specific sequences could affect the time of H3K4me3 deposition. A recent study reported that lysine-specific demethylase 1 (LSD1)-like histone demethylases (LDL1 and LDL2) can interact with CCA1/LHY to repress *TOC1* expression [59]. Moreover, LDL1 and LDL2 interact with HDA6 to co-regulate *TOC1* expression by histone demethylation and deacetylation. It is probable that this type of histone complex can include JMJ14 and other factors to regulate expression of clock genes. It is equally plausible that histone methyltransferases and acetyltransferases could be in the same complex to carry out the opposite direction of circadian-mediated gene expression. It will be interesting to further test how specific transcription factors including circadian clock regulators recruit other histone methyltransferases and demethylases to establish rhythmic histone modifications that exert growth and developmental regulation in plants and animals.

## Methods

### Plant materials and growth conditions

*A. thaliana* ecotypes Col-0 and Ws were used in this study. *cca1 lhy* mutant in Ws background and *CCA1-OX* transgenic lines in Col-0 background were generated as previously described [27]. The mutants *sdg2* (SALK-021008) and *jmj14* (SALK_135712) were obtained from Arabidopsis Biological Resource Center (ABRC). The primer sequences for genotyping are listed in Additional file 5: Table S4. For diurnal conditions, plants were grown under the light/dark (L/D) cycle of 16 h/L and 8 h/D at 22 °C.

### RNA extraction and qRT-PCR

Total RNA was isolated from aerial rosette leaves of ~ 3-week-old plants using Plant RNA Reagent (Thermo Fisher Scientific, Waltham, Massachusetts). After digestion by RNase-Free DNase (Promega, Madison, Wisconsin), total RNA (1 μg) was used to produce first-strand cDNA with the Omniscript RT Kit (Qiagen, Valencia, California). The cDNA was used as the template for qRT-PCR using FastStart Universal SYBR Green Master in a LightCycler®96 System (Roche, Indianapolis, Indiana). The relative expression level was quantified using the internal control *ACT7* (*AT5G09810*). Three biological replicates were performed for each experiment, and three technical replicates were used for each biological replicate in qRT-PCR analysis. Student’s *t* test and calculation of error bars were performed to determine the significance level in each comparison using three biological replicates. The primer sequences are listed in Additional file 5: Table S4.

### Histone extraction and Western blot analysis

Histone extraction was performed as described previously [60]. An aliquot (1 g) of aerial rosette leaves from ~ 3-week-old plants were ground to powder with liquid nitrogen and then suspended in NIB buffer (250 mM sucrose, 60 mM KCl, 15 mM NaCl, 5 mM MgCl_2_, 1 mM CaCl_2_, 15 mM PIPES, pH 6.8, 0.8% Triton X-100, 1 mM PMSF, and 1x cocktail). The homogenized solution was filtered through four layers of cheesecloth; the supernatant was centrifuged at 3000*g* for 20 min at 4 °C. Pellets were resuspended in 800 μl 0.4 N H_2_SO_4_ and incubated with a rotator overnight at 4 °C. After centrifugation at 16,000*g* at 4 °C for 10 min, the supernatant was transferred into a new tube by adding 264 μl trichloroacetic acid. Samples were incubated overnight at 4 °C and centrifuged again at 16,000g at 4 °C for 10 min to precipitate histone proteins. After washing by acetone, proteins were dissolved in water for Western blot analysis. The antibodies were anti-histone H3 (Abcam; Ab1791, 1:5000 dilution), anti-H3K4me3 (Abcam; Ab8580, 1:5000 dilution), and anti-H3K9Ac (Abcam; Ab10812, 1:5000 dilution).

### Chromatin immunoprecipitation (ChIP) and ChIP-seq

Chromatin immunoprecipitation was performed following the published protocol [61]. Briefly, 2 g of aerial rosette tissues from ~ 3-week-old *A. thaliana* (Col-0) were crosslinked in 1% formaldehyde, and the chromatin fractions were isolated and purified. Antibodies against histone H3 (Abcam; ab1791), H3K4me3 (Abcam; Ab8580), H3K9ac (Abcam; Ab10812), or CCA1 (Abiocode, R1234-3) were added to the chromatin extracts (with a 1:1000 dilution) and incubated overnight at 4 °C with gentle rotation. Immunoprecipitated DNA was extracted using Protein A/G Magnetic Beads (Thermo Fisher Scientific, Waltham, Massachusetts). Immunoprecipitated DNA (IP DNA) and input DNA (without IP but with incubation of beads) were subjected to reverse cross-linking and purified using QIAquick PCR Purification Kit (Qiagen, Valencia, California). For ChIP-seq, libraries with three biological replicates were constructed from IP DNA and non-IP DNA using NEBNext® Ultra™ II DNA Library Prep Kit for Illumina (NEB, Ipswich, Massachusetts). For ChIP-qPCR, immunoprecipitated DNA was used as the template for qPCR using FastStart Universal SYBR Green Master in a LightCycler®96 System (Roche, Indianapolis, Indiana). The relative enrichment was quantified using the internal control *AT2G26560*. Three biological replicates were performed for each experiment with three technical replicates for each biological replicate. Student’s *t* test was used to determine the significance level in each comparison.

### ChIP-seq data analysis

ChIP-seq dataset generated in this study and the published ChIP-seq datasets in *sdg2* and *jmj14* mutants were analyzed as follows. After removing adapters, low-quality reads, and PCR duplicates, the filtered pair-end reads were aligned to *A. thaliana* reference genome (TAIR10) using Bowtie2 (Version 2.3.0) with parameters “bowtie2 --no-mixed --no-discordant” [62]. Only uniquely and concordantly mapped reads were used for further analysis. In order to adjust different sequencing depths among different samples, the uniquely mapped reads were “down-sampled” to the lowest read number of samples, as previously described [56]. Enriched peaks were identified using Model-based Analysis of ChIP-Seq (MACS, version 2.1.0) with the option “macs2 callpeak -g dm -q 0.01” [63]. Peaks were used for further analysis only if they were present in two biological replicates. To make a master-peak list for all time points, the peaks obtained from each time point were merged. When different samples were compared, the read coverage was normalized to 1× sequencing depth to eliminate bias of different insertion lengths in pair-end reads. The genes overlapping with peaks in the gene body or ± 1-kb regions were defined as peak-overlapped genes. The peaks were compared between the wild type and *cca1 lhy* mutant using one-way ANOVA, and their significance was determined with *P* < 0.05. As no biological replicate was used from the ChIP-seq datasets in *sdg2* and *jmj14* mutants, we used 1.5-fold changes to determine differential peaks between the wild type and mutants. The non-parametric algorithm JTK_CYCLE was used to detect rhythmic peaks and identify the phase of each peak with *P*_adj_ < 0.01 [64]. Gene Ontology (GO) analysis was performed using DAVID [65].

## Additional files

Additional file 1: Supplementary Figure S1–S8. (PS 3131 kb)

Additional file 2: **Table S1.** List of peaks with different H3K4me3 enrichment between WT and *cca1 lhy* mutant. (XLSX 101 kb)

Additional file 3: **Table S2.** List of all identified H3K4me3 and H3K9ac peaks with coverage levels at all time points. (XLSX 11750 kb)

Additional file 4: **Table S3.** The list of diurnal H3K4me3 and H3K9ac peaks with coverage levels at all time points. (XLSX 2978 kb)

Additional file 5: **Table S4.** Primers used in this study. (DOCX 14 kb)

## Data Availability

All raw sequencing data have been deposited in NCBI Nucleotide and Sequence Read Archive (SRA) (https://trace.ncbi.nlm.nih.gov/Traces/sra/?study=SRP152291) [66]. Most analyses were performed using publicly available software, and the codes used for statistical analysis are available upon request.
