# Supplementary material for: Diurnal regulation of SDG2 and JMJ14 by circadian clock oscillators orchestrates histone modification rhythms in Arabidopsis
Source: Genome Biol. 2019 Aug 20;20:170. doi: 10.1186/s13059-019-1777-1 (PMC6892391; doi:10.1186/s13059-019-1777-1)
Supplement: Supplementary file 5 — Table S4. Primers used in this study. (DOCX 14 kb) [file 13059_2019_1777_MOESM5_ESM.docx]

| SALK-021008 | TCAGACGATGCTTATGGTTCC | Genotyping |
| --- | --- | --- |
|  | TTATGAACCAAAGCAAAACCG |  |
| SALK_135712 | GACAGGTGTACTATGGAGCTGACT |  |
|  | AGCAGATGGTTAGTACATAGCTCC |  |
| CCA1 | TCCAGATAAGAAGTCACGCTCAGA | ChIP-qPCR |
|  | CATTAAGCCAATGAAGATGAGAACA |  |
| SDG2 | TCCAGATCTGCATAAATCTCCT |  |
|  | TCAAAGCGTATATCTTGAGCGC |  |
| JMJ14 | TGGAGTCCGATCTAAGAAAAG |  |
|  | TATTTGGTATAAATGGATTGAAG |  |
| LHY | AATCTAAAGAGGTTATCACAACGGC |  |
|  | GCTGCTTCAAATCCTCTCTAACAAG |  |
| At2g26560 | GCTGCTACTCTTGCGTTCG |  |
|  | GCCTTAGCTGCCATCAAGG |  |
| JMJ14 | GGGCTTGAAATGTTTGGATTTCTC | RT-qPCR |
|  | CTTCAACAGTCCACGCAGAAGC |  |
| SDG2 | TTTAGCAGGTTGCCCTTTTG |  |
|  | AAAGCTGAGGACCTTTGCAG |  |
| CCA1 | AGGCTCGATCTTCACTGGACTCA |  |
|  | CCGCCTCAACATCATCACTACTCG |  |
| LHY | TGTGAACGGGAATATAGCAAAAT |  |
|  | TCTGGAGAAACGAACGGTAATCAT |  |

Table S4 Primers used in this study.
